# Neutralizing antibody response to Omicron subvariants BA.1 and BA.5 in children and adolescents following the two-dose CoronaVac protocol (Immunita-002, Brazil): a 12-month longitudinal study

**DOI:** 10.3389/fimmu.2025.1589733

**Published:** 2025-07-15

**Authors:** Camila Amormino Corsini, Guilherme Rodrigues Fernandes Campos, Priscila Fernanda da Silva Martins, Priscilla Soares Filgueiras, Ana Esther de Souza Lima, Sarah Vieira Contin Gomes, Caroline De Almeida Leitao Curimbaba, Daniela Aparecida Lorencini, Eolo Morandi Junior, Victor Mattos da Silva, Maria Célia Cervi, Marcos de Carvalho Borges, Poliana Remundini de Lima, João Paulo Resende do Nascimento, Paulo Roberto Lopes Correa, Leda dos Reis Castilho, Jaquelline Germano de Oliveira, Olindo Assis Martins Filho, Maurício Lacerda Nogueira, Rafaella Fortini Queiroz e Grenfell

**Affiliations:** ^1^ Instituto René Rachou, Oswaldo Cruz Foundation (FIOCRUZ), Belo Horizonte, Minas Gerais, Brazil; ^2^ Faculty of Medicine of São José do Rio Preto (FAMERP), São José do Rio Preto, São Paulo, Brazil; ^3^ Instituto Butantan, São Paulo, São Paulo, Brazil; ^4^ Faculty of Medicine, University of São Paulo (USP), São Paulo, São Paulo, Brazil; ^5^ Serrana Clinical Research Center, Serrana, São Paulo, Brazil; ^6^ Belo Horizonte Municipal Health Department (SMS), Belo Horizonte, Brazil; ^7^ Cell Culture Engineering Laboratory (COPPE), Federal University of Rio de Janeiro (UFRJ), Rio de Janeiro, Rio de Janeiro, Brazil; ^8^ Hospital de Base, São José do Rio Preto, São Paulo, Brazil; ^9^ Department of Pathology, University of Texas Medical Branch, Galveston, TX, United States; ^10^ Federal University of Minas Gerais (UFMG), Belo Horizonte, Minas Gerais, Brazil; ^11^ Department of Infectious Diseases, College of Veterinary Medicine, University of Georgia (UGA), Athens, GA, United States

**Keywords:** vaccine, covid-19, SARS-CoV-2, neutralizing antibody, Omicron, children and adolescents

## Abstract

**Introduction:**

The covid-19 pandemic prompted an unprecedented global effort to develop and deploy vaccines, including CoronaVac, an inactivated virus-based vaccine. While these vaccines effectively reduced severe cases and hospitalizations, limited data exists on their immunogenicity in younger populations, particularly children and adolescents. Understanding the immune response in these groups is essential to guide vaccination strategies and assess protection against emerging variants of concern, such as Omicron subvariants BA.1 and BA.5. This study evaluated the neutralizing antibody response in children and adolescents aged 3–17 years over 12 months following the two-dose CoronaVac protocol in Brazil.

**Methods:**

A cohort of 108 children (3–11 years) and adolescents (12–17 years) from Serrana, Brazil, received two doses of CoronaVac. Peripheral blood samples were collected at baseline, and at 1, 3, 6, and 12 months after the second dose. Participants were stratified by serostatus prior to vaccination. Neutralizing antibodies against Omicron BA.1 and BA.5 were assessed using microneutralization assays.

**Results:**

Neutralizing antibody titers increased significantly after vaccination in both seronegative and seropositive individuals. For seronegative participants, seroconversion rates for BA.5 rose from 16.6% pre-vaccination to 93.3% one month after the second dose in children, and from 50% to 92% in adolescents, with sustained levels for 12 months. Seropositive participants also showed enhanced antibody titers, particularly against BA.5. No significant differences in neutralization between BA.1 and BA.5 were observed post-vaccination, contrary to prior literature, suggesting uniform effectiveness against these subvariants.

**Discussion:**

This study demonstrates that CoronaVac significantly enhances and sustains neutralizing antibody titers in children and adolescents for up to one year, including against immune-evading subvariants like BA.5. The robust response highlights the vaccine’s potential as a critical tool for reducing SARS-CoV-2 transmission and preventing severe disease, particularly in regions with limited access to updated vaccines. Further studies with larger cohorts are needed to validate these findings and inform vaccination strategies for immunoresistant variants.

## 1 Introduction

For According to the World Health Organization (WHO), as of January 5, 2025, more than 777 million cases of COVID-19 had been confirmed worldwide. In Brazil, the number of confirmed cases surpassed 37 million, with approximately 702,000 deaths recorded by that date, making it the second country in terms of deaths from the disease, behind only the United States (1). In an unprecedented effort, covid-19 vaccines were rapidly developed and approved for emergency use, with notable examples including vaccines based on inactivated viruses, mRNA, and non-replicating adenoviral vectors (2). These vaccines demonstrated efficacy in reducing cases and deaths. However, the pandemic persisted due to the emergence and spread of SARS-CoV-2 variants characterized by higher transmissibility, infectivity, and the ability to evade both immunity induced by previous infections and immunity provided by available vaccines (3–5).

The inactivated virus vaccine platform used by CoronaVac has been shown to induce a robust immune response against various viral proteins, including the S (Spike), N (Nucleocapsid), and M (Membrane) proteins (6). Furthermore, CoronaVac has proven to be effective and safe, inducing high levels of neutralizing antibodies, with good tolerability and no severe adverse events or vaccine-related fatalities reported during clinical trials (7, 8). Its efficacy was reported as 83.5% against symptomatic COVID-19 among volunteers aged 18 to 59 years (7, 8).

By January 2022, approximately 85 million doses of this vaccine had been administered to the Brazilian population (9). In the same year, Anvisa (Brazil’s National Health Surveillance Agency) expanded the vaccination protocol to include children and adolescents nationwide (10). Although CoronaVac is no longer the primary vaccine used in Brazil, data from its widespread application continues to contribute to public health strategies worldwide (11). The immune response induced by COVID-19 vaccines remains under investigation, particularly in children and adolescents. In this age group, the duration and intensity of immune protection, as well as its efficacy against different variants of concern (VOCs), are not yet fully defined (11). These aspects are essential for determining the need for booster doses and supporting evidence-based decisions by healthcare managers (12). Based on this, the objective of the present study was to comprehensively evaluate the neutralizing antibody response in children and adolescents aged 3 to 17 years over 12 months following the administration of the primary two-dose CoronaVac protocol in Brazil against the Omicron subvariants BA.1 and BA.5 circulating in the country during 2022.

## 2 Methods

### 2.1 Ethics statement and participants

This study was approved by the Research Ethics Committee involving Human Subjects at the Oswaldo Cruz Foundation, the Ethics Committee of the Hospital das Clínicas of the Faculty of Medicine of Ribeirão Preto, University of São Paulo, and the National Council of Ethics in Research (CAAE 55183322.6.0000.5091). The study was supervised by the National Health Surveillance Agency.

Inclusion criteria included children and adolescents aged 3 to 17 years who were unvaccinated for covid-19 and who voluntarily participated in the study with the agreement of their parents or legal guardians, signing the informed consent and assent forms (ICF/IAF). Exclusion criteria included children and adolescents aged 6 to 17 years with immunosuppression, who were not eligible for participation. Additionally, children and adolescents who reported COVID-19 infection during the study were not included in the statistical analyses.

### 2.2 Participant recruitment, sample collection, and follow-up

Participants were invited to join the research at a public healthcare center located in Serrana, São Paulo, Brazil. A total of 108 participants who met the inclusion criteria were followed for twelve months after completing the two-dose primary protocol of the CoronaVac vaccine (Sinovac, Butantan Institute), administered with a 28-day interval between doses.

Peripheral blood samples were collected at multiple time points: prior to vaccination, on the day of the second dose administration, and at one, three, six, and twelve months post-second dose, relative to the date of administering the second dose of the CoronaVac vaccine (Sinovac, Butantan Institute). A 10 mL whole blood sample was obtained via venous puncture from each participant following biosafety standards and subsequently centrifuged at 3,000 g for 5 min to obtain serum for immunogenicity analyses. Samples were collected from March 2022 to July 2023.

### 2.3 Assessment of anti-S and anti-N IgG antibodies via ELISA for defining baseline seroreactivity

To assess baseline seroreactivity, enzyme-linked immunosorbent assays (ELISAs) were performed to detect IgG antibodies specific to the SARS-CoV-2 Spike (anti-S) and Nucleocapsid (anti-N) proteins. All serum samples obtained from the study participants were tested for total IgG antibodies specific to the Spike (S) and Nucleocapsid (N) proteins of SARS-CoV-2. Participants who tested reactive in both ELISA assays at the first time point of the study (detection of anti-S and anti-N IgG antibodies), before receiving the first dose of the vaccine, were classified as seropositive, while those who were non-reactive in both ELISA assays were classified as seronegative. These proteins, used as antigens, were derived from the Wuhan reference strain (B.1), and were generated in stable recombinant HEK293 cells, as described by Alvim et al. (2022) (13). Antibody detection was performed using standardized ELISA assays, following the methodology established by GRENFELL et al. (2022), which had been validated by the National Institute of Health Quality Control of the Oswaldo Cruz Foundation (INCQS/Fiocruz) (14). The cutoff value adopted for the determination of positivity was 0.1508. This cutoff value was previously established based on validated positive and negative controls. These controls were derived from samples of individuals with SARS-CoV-2 infection confirmed by RT-PCR, ensuring adequate sensitivity and specificity for the detection of IgG antibodies in the assay (14).

### 2.4 Viral neutralization assays to SARS-CoV-2 variants (BA.1 and BA.5)

All serum samples across all time points were subjected to neutralizing antibody assays (VNT50) to detect antibodies against the Omicron variant, subvariants BA.1 (HIAE –W.A) and BA.5 (EPI_ISL_18277186), as outlined by CAMPOS et al. (2022) (9). VNT50 was performed as published before (9, 11). Serum samples from children and adolescents were collected before and after vaccination, inactivated at 56°C for 30 minutes, and serially diluted two-fold (1:20 to 1:2560). Diluted samples were incubated for 1 hour at 37°C with 50 TCID50 of SARS-CoV-2 subvariants BA.1 and BA.5. After incubation, 100 µl of these solutions were transferred to Vero cell-seeded 96-well plates and incubated in supplemented DMEM for 72 hours at 37°C with 5% CO_2_. The median neutralization titer (VNT50) was determined as the reciprocal dilution providing 50% protection against cytopathic effects, calculated using the Spearman-Karber method. Each sample was tested in triplicate (15, 16). A dilution of 1:20 was established as the cutoff point for seroconversion.

### 2.5 Statistical analysis

Data analyses were performed using GraphPad Prism^®^ software version 8.0. The median neutralization titer (VNT50) was determined as the reciprocal of the dilution that provided 50% protection against cytopathic effects, calculated using the Spearman-Karber method. Antibody titer quantification results were analyzed statistically using the Kruskal-Wallis test, while pairwise comparisons were conducted using the Mann-Whitney test. A significance level of p < 0.05 was applied for all analyses. The correlation between neutralizing antibodies against BA.1 and BA.5 subvariants was evaluated using Spearman’s rank correlation coefficient, with statistical significance set at p < 0.05.

## 3 Results

### 3.1 Baseline characteristics of participants

In total, 108 individuals were included in this study, 60 (55.56%) children aged 3 to 11 years, and 48 adolescents aged 12 to 17 years (44.44%). For the seronegative group, 60 participants were included, and for the seropositive group, 48 participants were included, covering both age ranges. The remaining characteristics of the cohort, such as biological gender and comorbidities, are presented in **Table 1**.

**Table 1:** General characteristics of the included participants.

Epidemiological data	Seronegative before vaccination¹ (n,%)	Soropositive before vaccination² (n,%)	Total (n=108)
Age, years			
3-11	30, 27.78	30, 27.78	60, 55.56
12-17	30, 27.78	18, 16.67	48, 44.44
Biological gender			
Male	32, 29.63	18, 16.67	50, 46.30
Female	28, 25.93	30, 27.78	58, 53.70
Comorbidities			
Allergic rhinitis	3, 2.78	6, 5.56	9, 8.33
Asthma	1, 0.93	1, 0.93	2, 1.85
Obesity	0, 0	2, 1.85	2, 1.85
Hypothyroidism	0, 0	1, 0.93	1, 0.93
No comorbidities	56, 51.85	38, 35.19	94, 87.04

¹Seronegative for SARS-CoV-2 anti-S and anti-N IgG antibodies by ELISA prior to the CoronaVac primary vaccination protocol.

²Soropositive for SARS-CoV-2 anti-S and anti-N IgG antibodies by ELISA prior to the CoronaVac primary vaccination protocol.

### 3.2 Neutralization levels against BA.1 and BA.5 variants before and after vaccination

The viral microneutralization assay enabled the evaluation of seroconversion rates and the determination of mean neutralizing antibody titers against the Omicron subvariants BA.1 and BA.5 in children and adolescents over 12 months following the primary CoronaVac vaccination protocol.

**Figure 1:**
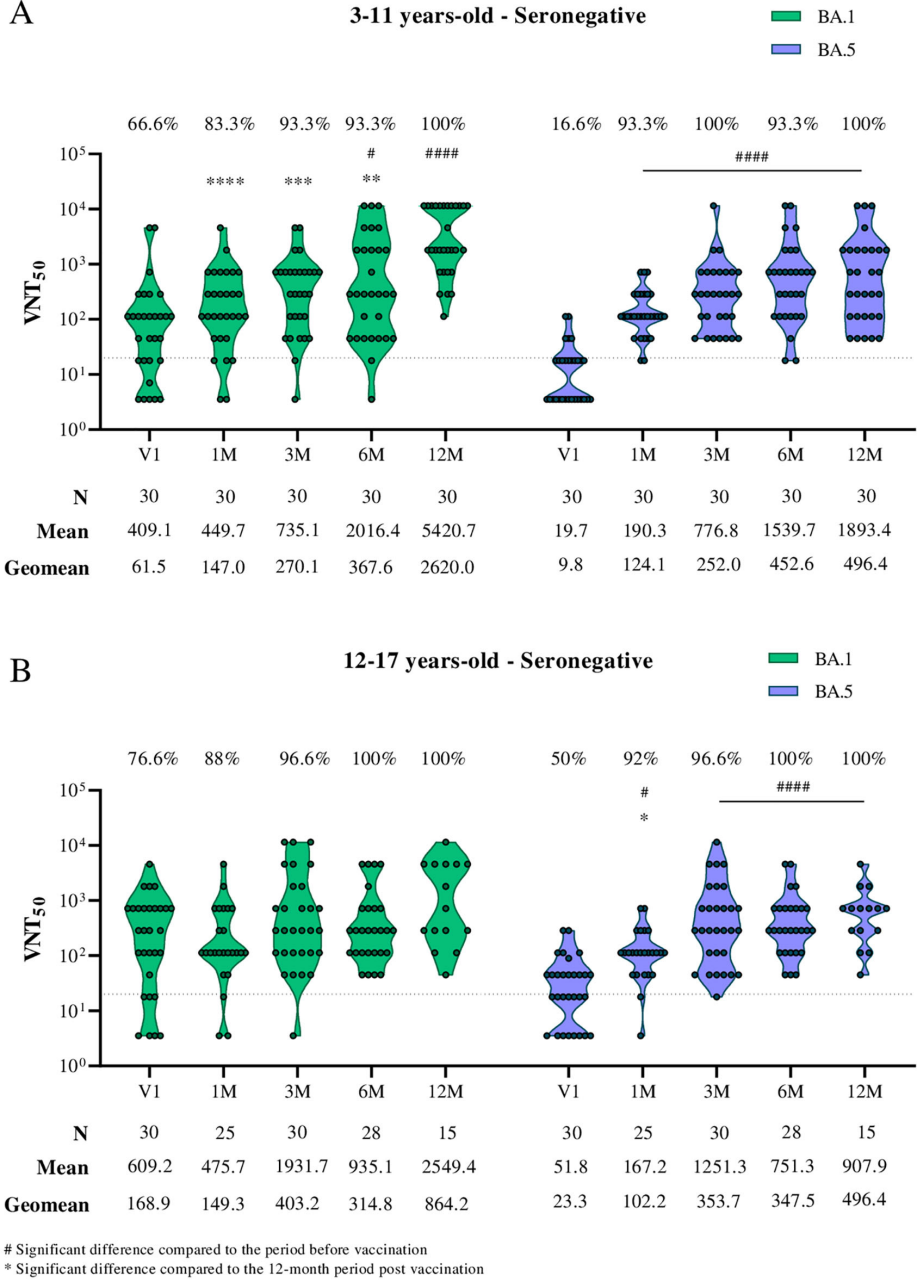
Viral microneutralization assay against Omicron subvariants to evaluate neutralization titers (VNT50) and seroconversion rates over 12 months in children and adolescents vaccinated with the CoronaVac primary protocol. The Omicron subvariants BA.1 and BA.5 are represented in green and purple, respectively. **(A)** Neutralizing antibodies in children aged 3 to 11 years seronegative for SARS-CoV-2 S and N antibodies before the CoronaVac primary protocol. **(B)** Neutralizing antibodies in adolescents aged 12 to 17 years seronegative for SARS-CoV-2 S and N antibodies before the CoronaVac primary protocol. The sample size (n), VNT50 means, and geometric mean titers for each group are highlighted below the graphs. Dashed lines represent the seroconversion dilution cutoff (1:20), while seroconversion rates are expressed as percentages. Significance lines indicate differences among the mean neutralization titers of the groups. P-values lower than 0.05 were considered significant.

In the evaluation of neutralizing antibodies in seronegative individuals, a significant increase in antibody titers was observed after the primary CoronaVac vaccination protocol, both for the BA.1 and BA.5 subvariants, in children aged 3 to 11 years (**Figure 1A**). Notably, seropositivity for the BA.5 subvariant increased from 16.6% prior to vaccination to 93.3% one month after the second vaccine dose and remained high up to the last follow-up point (12 months post-second dose).

In the evaluation of seronegative adolescents (aged 12–17; **Figure 1B**), no significant difference in neutralizing antibody titers against the BA.1 subvariant was observed after the primary vaccination protocol. However, for the BA.5 subvariant, seropositivity increased significantly from 50% to 92% after vaccination, remaining elevated until the study’s last follow-up point.

**Figure 2:**
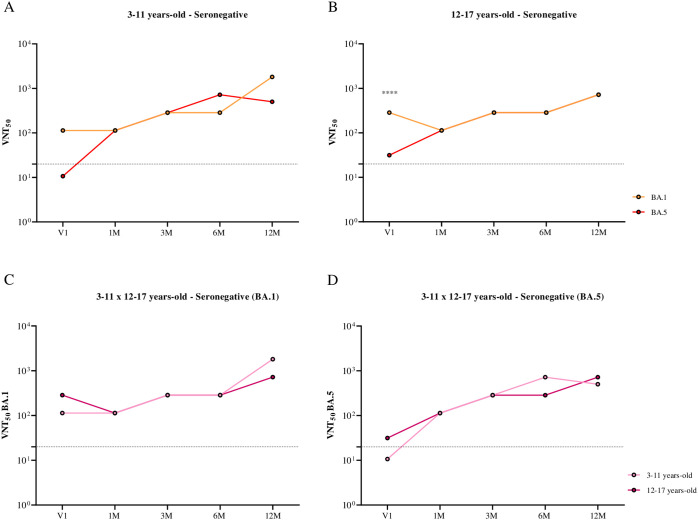
Comparison of the kinetics of neutralizing antibodies against BA.1 and BA.5, stratified by age group and subvariant, in children and adolescents seronegative for SARS-CoV-2 S and N antibodies before the CoronaVac primary protocol, over 12 months following the administration of two doses of the CoronaVac vaccine. **(A)** Comparison of the kinetics of neutralizing antibodies against BA.1 and BA.5 in children aged 3 to 11 years. **(B)** Comparison of the kinetics of neutralizing antibodies against BA.1 and BA.5 in adolescents aged 12 to 17 years. **(C)** Comparison of the kinetics of neutralizing antibodies against the BA.1 subvariant in children and adolescents aged 3 to 17 years. **(D)** Comparison of the kinetics of neutralizing antibodies against the BA.5 subvariant in children and adolescents aged 3 to 17 years. Dashed lines represent the seroconversion dilution cutoff (1:20), while seroconversion rates are expressed as percentages. Significance lines indicate differences among the mean neutralization titers of the groups. P-values lower than 0.05 were considered significant.

When comparing neutralizing antibody titers against BA.1 and BA.5 separately by age group (**Figures 2A, B**) and by subvariant (**Figures 2C, D**) in seronegative individuals, higher antibody titers against BA.1 were observed in adolescents before receiving the first dose of CoronaVac (V1). This finding may indicate prior infection with this subvariant in this group.

**Figure 3:**
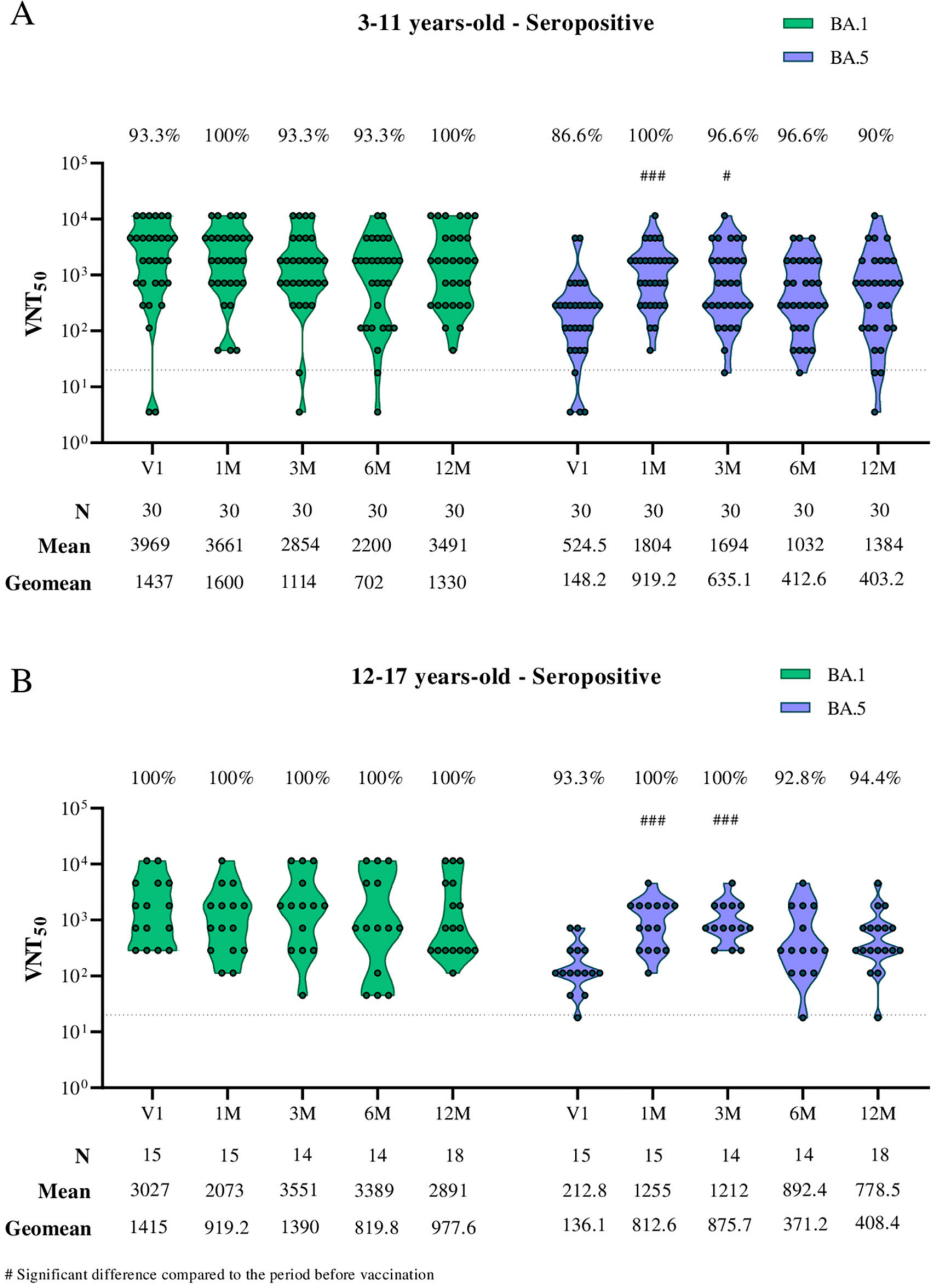
Viral microneutralization assay against Omicron subvariants to evaluate neutralization titers (VNT50) and seroconversion rates over 12 months in children and adolescents vaccinated with the CoronaVac primary protocol. The Omicron subvariants BA.1 and BA.5 are represented in green and purple, respectively. **(A)** Neutralizing antibodies in children aged 3 to 11 years seropositive for SARS-CoV-2 S and N antibodies before the CoronaVac primary protocol. **(B)** Neutralizing antibodies in adolescents aged 12 to 17 years seropositive for SARS-CoV-2 S and N antibodies before the CoronaVac primary protocol. The sample size (n), VNT50 means, and geometric mean titers for each group are highlighted below the graphs. Dashed lines represent the seroconversion dilution cutoff (1:20), while seroconversion rates are expressed as percentages. Significance lines indicate differences among the mean neutralization titers of the groups. P-values lower than 0.05 were considered significant.

In the evaluation of neutralizing antibodies in seropositive individuals prior to receiving the primary protocol, a significant increase in neutralizing antibody titers post-CoronaVac vaccination was observed only for the BA.5 subvariant. Seropositivity increased from 86.6% to 100% in children aged 3 to 11 years (**Figure 3A**) and from 93.3% to 100% in adolescents aged 12 to 17 years (**Figure 3B**).

**Figure 4:**
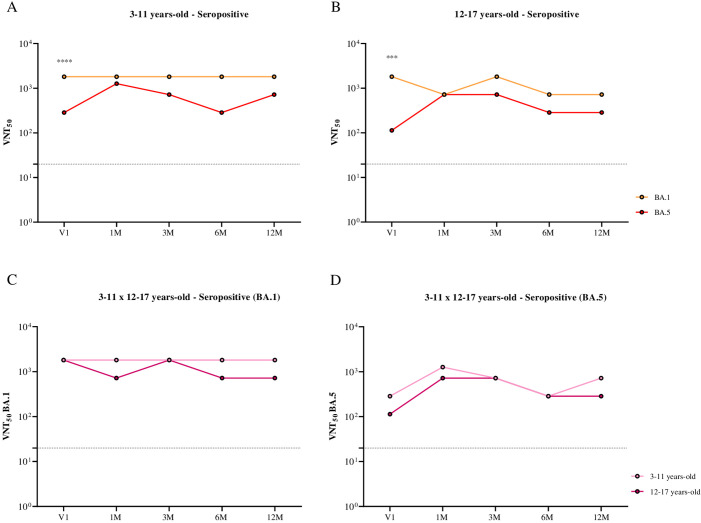
Comparison of the kinetics of neutralizing antibodies against BA.1 and BA.5, stratified by age group and subvariant, in children and adolescents seropositive for SARS-CoV-2 S and N antibodies before the CoronaVac primary protocol, over 12 months following the administration of two doses of the CoronaVac vaccine. **(A)** Comparison of the kinetics of neutralizing antibodies against BA.1 and BA.5 in children aged 3 to 11 years. **(B)** Comparison of the kinetics of neutralizing antibodies against BA.1 and BA.5 in adolescents aged 12 to 17 years. **(C)** Comparison of the kinetics of neutralizing antibodies against the BA.1 subvariant in children and adolescents aged 3 to 17 years. **(D)** Comparison of the kinetics of neutralizing antibodies against the BA.5 subvariant in children and adolescents aged 3 to 17 years. Dashed lines represent the seroconversion dilution cutoff (1:20), while seroconversion rates are expressed as percentages. Significance lines indicate differences among the mean neutralization titers of the groups. P-values lower than 0.05 were considered significant.

When comparing neutralizing antibody titers against BA.1 and BA.5 separately by age group (**Figures 4A, B**) and by subvariant (**Figures 4C, D**) in seropositive individuals, higher neutralizing antibody titers against the BA.1 subvariant were observed in both children and adolescents before receiving the first dose of CoronaVac (V1). This finding may also suggest prior infection with this subvariant in these groups.

### 3.3 Correlation between neutralizing antibodies against BA.1 and BA.5 variants

**Figure 5:**
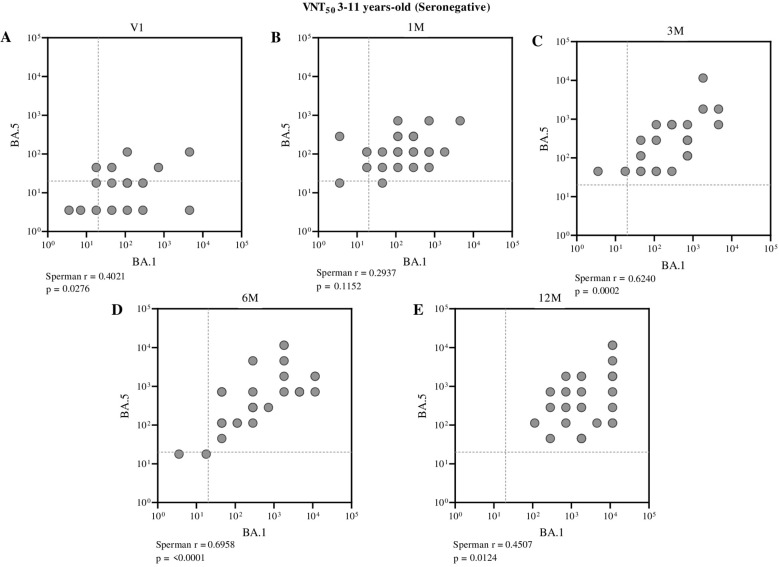
Correlation between neutralizing antibody titers against BA.1 and BA.5 in seronegative children (aged 3 to 11 years), assessed at different time points: before vaccination **(A)**, one month **(B)**, three months **(C)**, six months **(D)**, and twelve months **(E)** after receiving the second dose of CoronaVac. Each point represents an individual sample from a participant. The assay cutoff value of 20 is indicated by the dashed lines on the x and y axes. Spearman’s correlation coefficient was used, with statistical significance set at p < 0.05.

**Figure 6:**
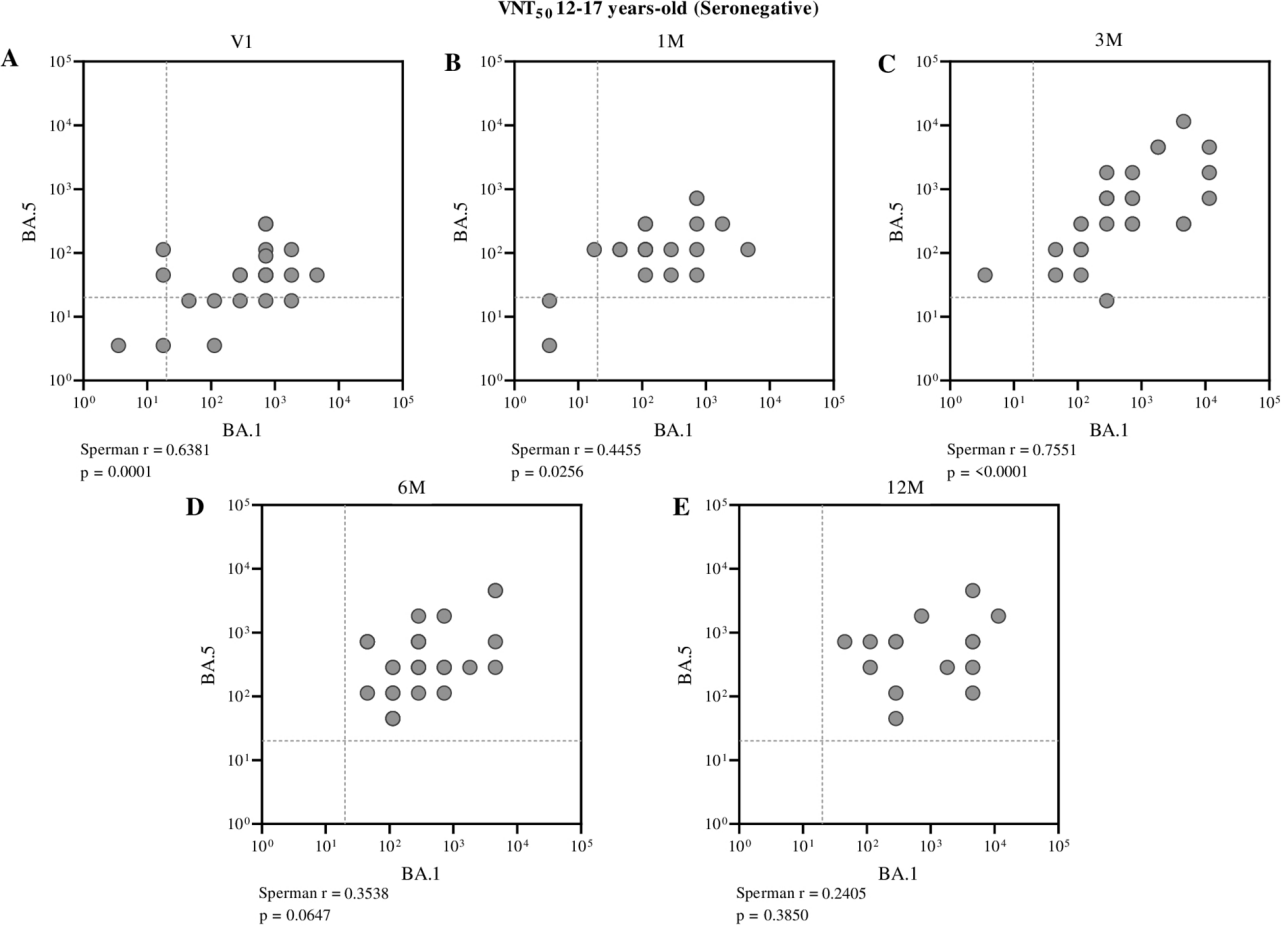
Correlation between neutralizing antibody titers against BA.1 and BA.5 in seronegative adolescents (aged 12 to 17 years), assessed at different time points: before vaccination **(A)**, one month **(B)**, three months **(C)**, six months **(D)**, and twelve months **(E)** after receiving the second dose of CoronaVac. Each point represents an individual sample from a participant. The assay cutoff value of 20 is indicated by the dashed lines on the x and y axes. Spearman’s correlation coefficient was used, with statistical significance set at p < 0.05.

When evaluating the correlation between neutralizing antibodies against BA.1 and BA.5 in seronegative children by ELISA, a moderate and statistically significant positive correlation was observed in most time points analyzed after vaccination (**Figure 5**). In adolescents, a strong positive correlation was identified at the first pre-vaccination time point (Spearman r = 0.6381, p = 0.0001) and further intensified three months after receiving the second dose of CoronaVac (Spearman r = 0.7551, p < 0.0001), suggesting a consistent association between these parameters (**Figure 6**).

**Figure 7:**
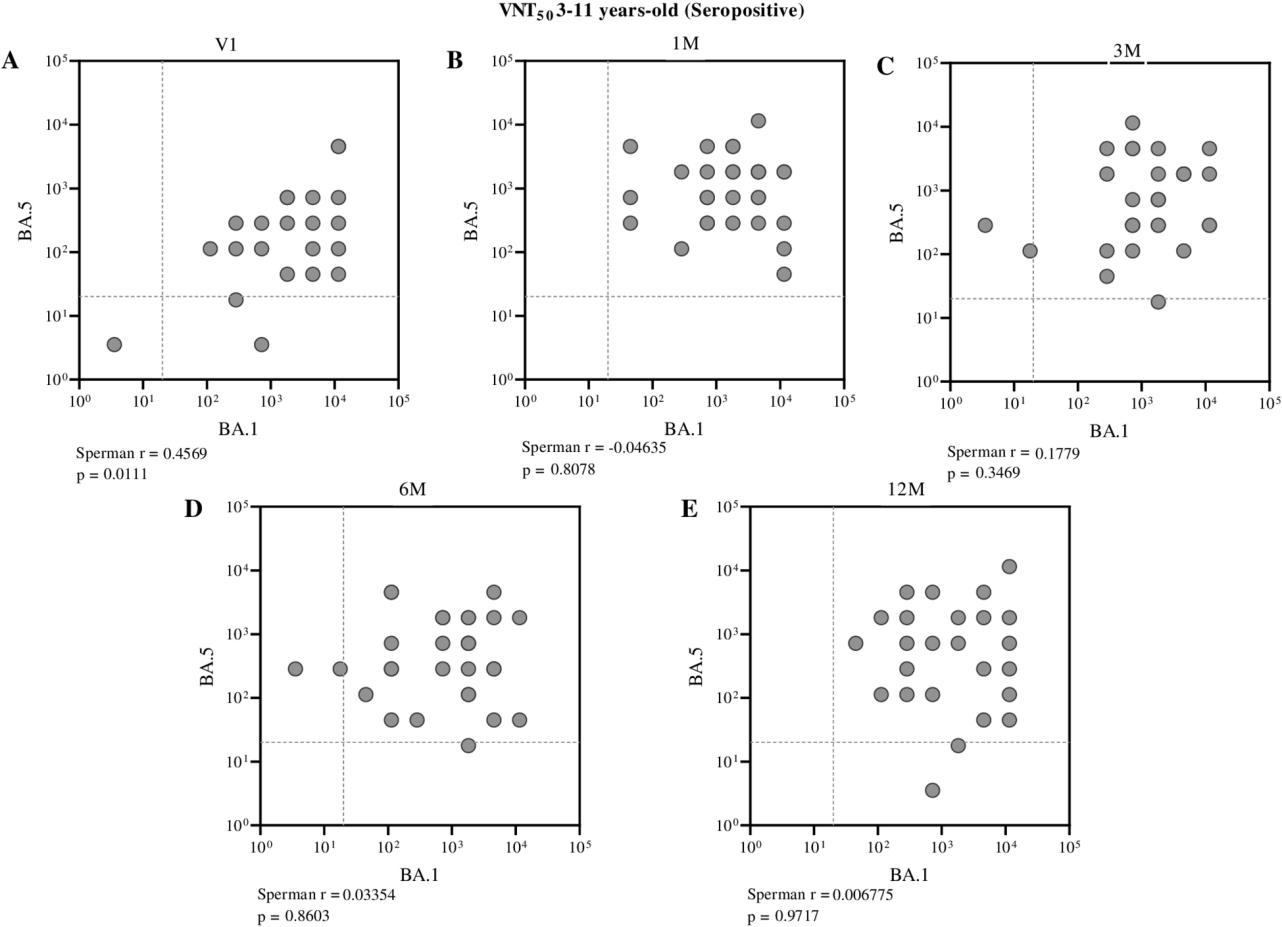
Correlation between neutralizing antibody titers against BA.1 and BA.5 in seropositive children (aged 3 to 11 years), assessed at different time points: before vaccination **(A)**, one month **(B)**, three months **(C)**, six months **(D)**, and twelve months **(E)** after receiving the second dose of CoronaVac. Each point represents an individual sample from a participant. The assay cutoff value of 20 is indicated by the dashed lines on the x and y axes. Spearman’s correlation coefficient was used, with statistical significance set at p < 0.05.

**Figure 8:**
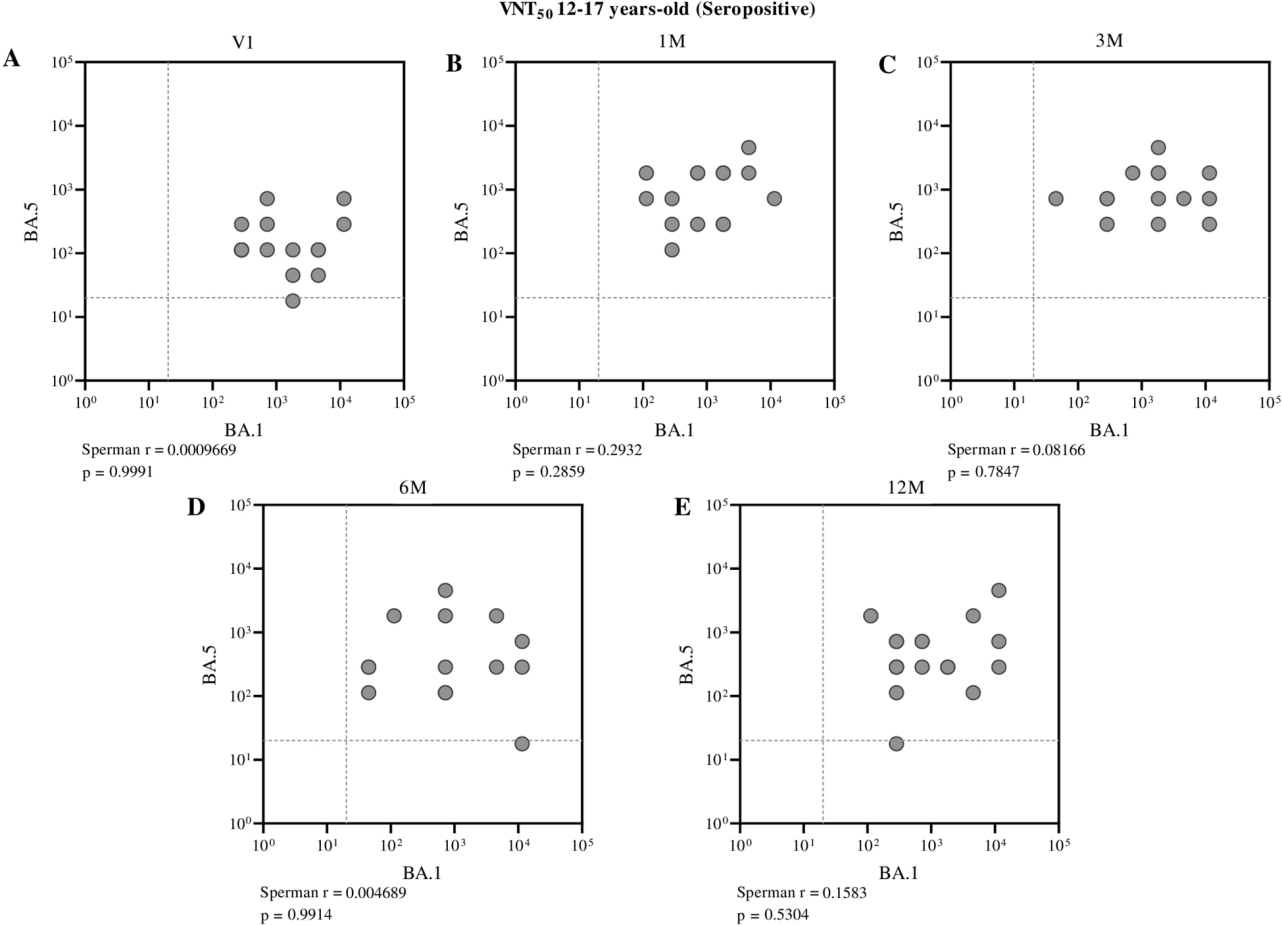
Correlation between neutralizing antibody titers against BA.1 and BA.5 in seropositive adolescents (aged 12 to 17 years), assessed at different time points: before vaccination **(A)**, one month **(B)**, three months **(C)**, six months **(D)**, and twelve months **(E)** after receiving the second dose of CoronaVac. Each point represents an individual sample from a participant. The assay cutoff value of 20 is indicated by the dashed lines on the x and y axes. Spearman’s correlation coefficient was used, with statistical significance set at p < 0.05.

Conversely, among seropositive individuals, only a moderate correlation between these neutralizing antibodies was observed before the administration of the first dose (Spearman r = 0.4569, p = 0.0111) in children aged 3 to 11 years (**Figure 7**). In adolescents, however, no significant correlation was found between BA.1 and BA.5 neutralizing antibodies at pre- and post-vaccination time points, indicating a weak or nonexistent association within this group across the evaluated periods (**Figure 8**).

## 4 Discussion

The COVID-19 pandemic reshaped immunization strategies, accelerating vaccine development and distribution to curb viral spread and new variants. Global collaboration among institutions, scientists, and regulatory agencies enabled the rapid rollout of safe and effective vaccines, allowing mass immunization within a year of the pandemic’s onset, significantly reducing cases, hospitalizations, and deaths (7, 17–25).

Initially, phase II and III clinical trials prioritized adults and the elderly, as they were the most affected (18, 21). Consequently, vaccines were first approved for adults, while children and adolescents relied on non-pharmacological measures during early waves of infection (26–28). Clinical trials for younger populations began later, following safety and efficacy data from adult studies (29–31). As mass vaccination advanced, younger age groups gained attention, particularly during the Delta variant surge, when adults and the elderly were fully immunized, leaving individuals under 18 as the most exposed group (32, 33). Beyond direct clinical impacts, their lack of immunization sustained viral transmission, potentially contributing to new variant emergence (34).

This shift in vaccination priority diverged from traditional immunization programs, such as in Brazil, where most vaccines are administered within the first 15 months of life to ensure early protection (35, 36). However, prioritizing high-risk groups—elderly individuals with immune senescence and middle-aged adults with frequent exposure—was a logical and effective approach, demonstrating success in controlling the pandemic (23, 37–40).

A key concern regarding childhood and adolescent immunization was the potential herd immunity from prior SARS-CoV-2 exposure. The delayed vaccination in this group led to increased infections, resulting in a significant number of individuals with prior virus contact. This background informed the study’s design, distinguishing groups based on confirmed previous infection.

Our data shows that neutralizing antibodies against Omicron variants, especially BA.1, were also detected prior vaccination in children and adolescents without history of previous infection, indicating the occurrence of asymptomatic cases. Some studies highlight that youngsters, when compared to adults and elderly people, are more likely to develop asymptomatic infections (41, 42), and this scenario represents a great challenge in determining the real infectiousness of this age group since these infections are mostly under-reported (43, 44).

On the other hand, despite neutralizing antibodies being detected in seropositive and seronegative groups prior vaccination, our results suggest an important contribution of immunization in the improvement of serological response. When compared to V1 (time-point before immunization), neutralization titers were significantly enhanced in children and adolescents by CoronaVac vaccination, especially against BA.5 subvariant. These data corroborate with some studies in the literature, where COVID-19 vaccination significantly improved the antibody response in individuals previously infected by SARS-CoV-2 when compared to vaccinated naïve individuals (45–47). Additionally, it is described that this hybrid immunity also showed higher serological protection in the respiratory tract, the main infection route of SARS-CoV-2, especially due to elevated levels of IgA antibody response in the mucosa after vaccination (48, 49).

A similar pattern was observed in individuals vaccinated with CoronaVac, the same immunizing platform used in this study. A study performed by Niyomnaitham et al., in 2022, evaluated the impact of different vaccines in naïve and previously infected participants. As expected, CoronaVac showed lower responses when compared to other vaccines, but using a SARS-CoV-2 pseudo virus neutralization assay, the authors observed that a single dose of CoronaVac was able to induce the same neutralization titer, against Omicron variant, as naïve individuals vaccinated with two doses of BNT162b2 (50). Our results corroborate and reinforce this observation, since we performed all neutralization assays using infectious particles instead of pseudo virus platform, showing that a two doses immunization with CoronaVac was capable to enhance and maintain high levels of antibody response against Omicron subvariants, independently of previous contact with SARS-CoV-2.

Other vaccination platforms, using attenuated adenoviral vector or mRNA as the immunizing agent, presented the same trend on improving immune protection (51, 52). A study performed in the United Kingdom, conducted with more than 35 thousand asymptomatic healthcare workers, showed that both serological and cellular immunity acquired only by previous infection decay after 1 year. However, after full vaccination of these seropositive individuals with ChAdOx1 nCoV-19 or BNT162b2 vaccines, protection levels remained high and consistent over time (90% of effectiveness on preventing subsequent infections) (53).

From the serological response perspective, our findings highlight that vaccination of children and adolescents, with CoronaVac, induced high levels of neutralizing antibodies against BA.1 and BA.5, two Omicron subvariants with different immunological features that influence on neutralization escape (54). Some studies, including a previous one from our group, showed that BA.5 is less neutralized by previous infection (55) and vaccination induced antibodies (11, 56, 57) than BA.1, the first omicron subvariant that emerged.

In this study, the correlation between neutralizing antibodies against BA.1 and BA.5 in seronegative children and adolescents, revealing a moderate and statistically significant positive correlation at most time points analyzed post-vaccination. Additionally, interestingly, the data presented here showed that, when comparing neutralization levels against BA.1 and BA.5 in children and adolescents, no significant differences were observed after CoronaVac administration, and this response was maintained over time. This finding contrasts with previous reports from *in vitro* and cohort studies, which suggest differential neutralization efficacy against these subvariants (58, 59). As an example, a study conducted in Japan with 13 thousand individuals, during BA.1/BA.2 and BA.5 infection waves, showed that vaccination protection against BA.5 was short-lasting and probably contributed to BA.5 infection peak (60).

This induction and maintenance of considerable titers of neutralizing antibodies in both age groups, independently of infection history, could suggest new perspectives on vaccination protocols for immunoresistant subvariants such as BA.5.

In a scenario where SARS-CoV-2 continues to circulate and evolve, updated monovalent vaccines, specifically targeting currently circulating variants, have replaced the previous bivalent Wuhan/BA.5 vaccines and are now considered essential tools. Some of these updated vaccines have already been tested and approved (61–65). Although CoronaVac is no longer the primary vaccine used in Brazil, the accumulated data from its widespread application continue to inform public health strategies globally. Moreover, CoronaVac, as a safe and effective inactivated virus vaccine, remains a valuable tool for controlling SARS-CoV-2 infection and preventing progression to severe disease, particularly in countries where updated vaccines are not yet readily available (11).

As a limitation of our study, since our data showed no difference on neutralization titers, after immunization, between subvariants, and this opposes the literature regarding BA.5 immune escape, an increase in the number of samples could strengthen even more the findings about vaccination and protection of youth population. As a methodological limitation of the study, regarding the criterion adopted to define prior SARS-CoV-2 infection, based on simultaneous positivity for anti-S and anti-N IgG antibodies detected by ELISA using antigens from the Wuhan reference strain, although this approach was chosen to ensure greater specificity, it is possible that it led to the misclassification of some previously infected individuals as seronegative. This misclassification may result from both the natural waning of total antibody levels over time and the attenuated immune response induced by variants such as Omicron, which may elicit antibodies with low affinity for ancestral strain antigens, thus hindering their detection by ELISA-based assays. The presence of relatively high neutralizing antibody titers in some participants classified as seronegative prior to vaccination reinforces this possibility, suggesting the occurrence of asymptomatic infections that were not serologically detected. Therefore, we acknowledge that this approach may have underestimated the proportion of individuals with prior infection, which should be considered when interpreting the immunological results observed after vaccination. In addition, it is important to consider that the neutralization assays performed in this study used total serum samples, without prior separation by immunoglobulin isotype. Therefore, it is not possible to attribute the observed neutralizing activity exclusively to the IgG fraction. Other isotypes, such as IgM and especially IgA, the latter particularly relevant in mucosal immune responses, may have contributed to the detected neutralization titers, particularly during the early stages of the immune response following vaccination. This potential interference should be considered when interpreting the data, as the total neutralizing activity measured does not necessarily reflect only the long-term humoral memory response mediated by IgG.

The results presented here highlight important and necessary information regarding vaccination of children and adolescents. A full immunization protocol with CoronaVac contributed to a significant enhancement of serological response for naïve and previous infected individuals, including against immunoresistant subvariants such as BA.5, and this robust antibody neutralization is stable for one year after vaccination. This positive response, in a population that was vaccinated later, could be crucial to deaccelerate SARS-CoV-2 circulation and reduce the emergence of new subvariants. In addition, an inactivated viral vaccine showed to be an interesting tool to increase immunity of less protected individuals, especially in regions where new and updated vaccines are not available yet.

## Data Availability

The raw data supporting the conclusions of this article will be made available by the authors, without undue reservation.

## Group members of Immunita team

Ana Clara Almeida^1^, Daniel Alvim Pena de Miranda^1^, Isabela Camargos^1^, Raquel Amorim^1^, Érica Louback de Oliveira^1^, Clara Raíssa do Prado Pêgo^1^, Viviane Cristina Fernandes dos Santos^1^.
